# SnO_2_-Based Interfacial Engineering towards Improved Perovskite Solar Cells

**DOI:** 10.3390/nano14171406

**Published:** 2024-08-28

**Authors:** Bing’e Li, Chuangping Liu, Xiaoli Zhang

**Affiliations:** Guangdong Provincial Key Laboratory of Information Photonics Technology, School of Physics and Opto-Electronic Engineering, Guangdong University of Technology, Guangzhou 510006, China; 2112315061@mail2.gdut.edu.cn (B.L.); 2112315025@mail2.gdut.edu.cn (C.L.)

**Keywords:** perovskite solar cells, aqueous SnO_2_, interfacial engineering

## Abstract

Interfacial engineering is of great concern in photovoltaic devices. Metal halide perovskite solar cells (PSCs) have garnered much attention due to their impressive development in power conversion efficiencies (PCEs). Benefiting from high electron mobility and good energy-level alignment with perovskite, aqueous SnO_2_ as an electron transport layer has been widely used in n-i-p perovskite solar cells. However, the interfacial engineering of an aqueous SnO_2_ layer on PSCs is still an obscure and confusing process. Herein, we proposed the preparation of n-i-p perovskite solar cells with different concentrations of SnO_2_ as electron transport layers and achieved optimized PCE with an efficiency of 20.27%. I Interfacial engineering with regard to the SnO_2_ layer is investigated by observing the surface morphology, space charge-limited current (SCLC) with the use of an electron-only device, and time-resolved photoluminescence (TRPL) of perovskite films.

## 1. Introduction

Named after mineral calcium titanate (CaTiO_3_), metal halide perovskite originally possessed the same crystal structure as that of the former, with the chemical formula ABX_3_. The A-site cation is coordinated to 12 X anions, forming a cuboctahedron, while the six-fold-coordinated B-site cation has an octahedral geometry [1,2,3]. Metal halide perovskite-based solar cells have garnered much attention worldwide due to the rapid progress they have facilitated in power conversion efficiency (PCE), and they have become a potentially strong competitor in the photovoltaic performance race [4,5,6]. Moreover, PSCs exhibit great promise in large-scale production and mainstream technology, owing to their low-cost, scalable, and simple solution processing techniques [7,8,9]. Despite the tremendous breakthroughs and rapid progress in the photovoltaic performance of perovskite solar cells, from 9.7% with pure MAPbI_3_ in 2012 to 26%, the remaining issues in interfacial engineering that urgently need to be resolved constitute a multifaceted challenge [10,11,12].

The n-i-p PSC configuration is regarded as a normal device structure; the active perovskite layer is directly spin-coated on an n-type electron transport layer (ETL), such as SnO_2_. The crystalline quality of the perovskite and the morphology of the buried interface are directly affected by the surface chemistry of the ETL [13,14,15]. Therefore, research on ETL has become one of the most relevant scientific subjects in the development of highly efficient and stable n-i-p PSCs. For instance, Yan et al. reported low-temperature solution-processed nanocrystalline SnO_2_ as an excellent alternative ETL and demonstrated PSCs with an average efficiency of 16.02% [16]. Kim et al. studied the band alignment between La-doped BaSnO_3_ (LBSO) and MAPbI_3_ perovskite, demonstrating LBSO as the next-generation ETL, with its high mobility, photostability and structural stability [17]. Pang and co-workers designed a Cl-containing tin-based ETL, SnO_x_-Cl, to realized a spontaneous ion exchange reaction at the interface of SnO*x*–Cl/MAPbI_3_, producing PSCs with an efficiency of 20.32% [18]. Chlorine-capped TiO_2_ colloidal nanocrystals were applied in a PSC to mitigate interfacial recombination and improve interface binding [19].

Aqueous SnO_2_ has been widely employed in n-i-p perovskite solar cells due to its high electron mobility and good energy-level alignment with perovskite and electrodes [20,21,22,23,24]. A SnO_2_ ETL can be obtained via the thermal oxidation of Sn(iv) isoprenoids, SnO_2_ quantum dots, and ALD (atomic layer deposition) [25,26,27,28]. Theories on the formation and properties of crystalline interfaces have been developed [29,30]. An optimized SnO_2_ electron transport layer showed great advantages, in terms of reduced interfacial recombination losses, controlled energy levels, and increased charge transport. SnO_2_ purchased directly from the market requires concentration dilution before application in PSCs, but the optimization of SnO_2_ concentrations is rarely reported. Moreover, researchers usually determine dilute SnO_2_ concentration ratios experientially. For example, Yang and co-workers reported a SnO_2_ precursor diluted in isopropanol and deionized water in a ratio of 1:3:2.5, achieving the same PCE as that of flexible PSC, reaching up to 18.71% [31]. Zhou et al. employed a 15 wt.% SnO_2_ aqueous solution in H_2_O as an ETL and realized a PCE of 21.92% [32]. However, those reports reached no consensus on the optimal SnO_2_ dilution ratio. At the same time, fundamental knowledge is lacking for a thorough understanding of the key role of different concentrations of SnO_2_ in crystallisation kinetics. Therefore, it is necessary to further explore the effects of concentrations of SnO_2_ as an ETL on device performance.

Here, we propose a simple and effective strategy to adjust the concentration of SnO_2_ and preliminarily validate the optimal dilution ratio of SnO_2_ in water. In this work, the efficiency of the device prepared by using the optimal concentration ratio (SnO_2_ 2.4%) reached 20.27%. This work provides a comprehensive understanding of SnO_2_ concentrations and of how to realize efficient perovskite photovoltaic devices with optimized SnO_2_ layers.

## 2. Experimental Section

*Materials*. All chemicals and materials were purchased from Aladin and directly used in the reactions and depositions without further purification. SnO_2_ hydrocolloidal dispersion solution (SnO_2_, 99.99%), leaching iodide (PbI_2_, 99.99%), lead bromide (PbBr2, 99.99%), formamidine hydroiodide (FAI, 99.99%), formamidine bromide (FABr, 99.99%), cesium iodide (CsI, 99.99%), methylammonium iodide (MAI, 99.99%), methylammonium chloride (MACl, 99.99%), n, n-dimethylformamide (DMF, 99.99%), dimethyl sulfoxide (DMSO, 99.99%), ethyl acetate (EA, 99.99%), Spiro-OMeTAD (99.99%), LiTFSi (99.99%), and 4-tert-butylpyridine (tBP, 99.99%) were used.

*Device Fabrication*. The FTO substrate was pre-washed with pure water, acetone, and isopropanol in an ultrasound bath for 15 min, followed by undergoing UV zone treatment for 20 min. We prepared four kinds of perovskite solar cell devices with different concentrations of SnO_2_. SnO_2_ was spin-coated at a speed of 3000 r/30 s, followed by undergoing annealing at 200 °C for 40 min. The perovskite film was deposited onto an electron transport layer, followed by the preparation of a hole transport layer (HTL) and a Ag metal electrode. Finally, devices with a structure of FTO/ETL/Perovskite/HTL/Ag were fabricated.

*Measurement.* The current density–voltage (J–V) characteristics of the perovskite solar cells were measured using an integrated solar simulator (JIS C 8942 Class MA). Solar cell performance was characterized under illumination using a standard amorphous Si photodetector (BS 520 S/N 007, Bunko Keiki, Tokyo, Japan), an air mass 1.5 global (AM 1.5 G) solar simulator with an irradiation intensity of 100 mw/cm^2^. Furthermore, 0.1 cm^2^ size apertures made of thin metal were attached to each cell before measurement. Scanning electron microscopy (SEM) was performed using JSM-7800F to analyze the surface morphology of the perovskite thin films.

## 3. Results and Discussion

**Figure 1:**
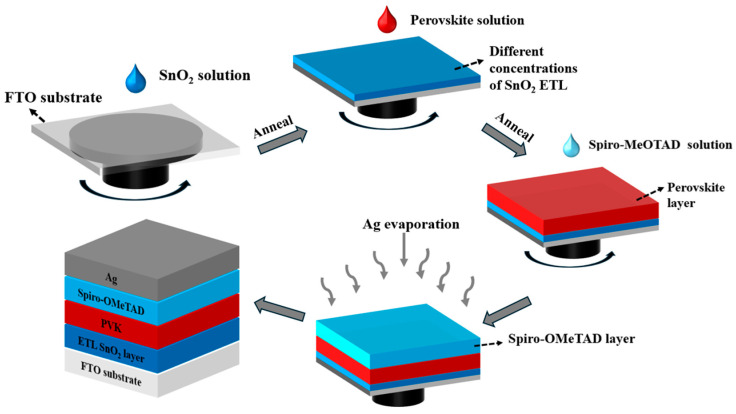
Schematic illustration of device fabrication process.

A plane structure diagram of an n-i-p perovskite solar cell composed of FTO/ETL/Perovskite/Spiro-OMeTAD/Ag is shown in Figure 1, and the detailed fabrication process is displayed accordingly. It is obvious that the n-i-p perovskite solar cell consists of the conductive substrate FTO, a SnO_2_ electron transport layer, a perovskite active layer, a Spiro-OMeTAD hole transport layer, and a conductive metal electrode made of Ag. The SnO_2_ ETL was employed to transport electrons that were generated in the active perovskite to the circuit. Therefore, systematic studies of SnO_2_ electron transport layers are of critical importance for the development of perovskite solar cells. 

In this work, the original aqueous SnO_2_ (12%) purchased from the aforementioned company was employed after further processing. In detail, the original SnO_2_ was in the form of nanoparticles, which were subjected to dilution with water, forming SnO_2_ concentrations of 4%, 3%, 2.4%, and 2%. The SnO_2_ solutions with varying degrees of dilution showed varied surface characteristics, directly affecting perovskite crystallization. Based on this, the surface morphology evolution and conductivity of the perovskite layer deposited on SnO_2_ thin films with different concentrations and the performance variation of the PSCs based on different SnO_2_ concentrations were studied. Interfacial engineering based on SnO_2_ ETLs was discussed systematically, and this is expected to provide in-depth understanding of ETL dynamics and give guidance on enhanced PSC performance.

**Figure 2:**
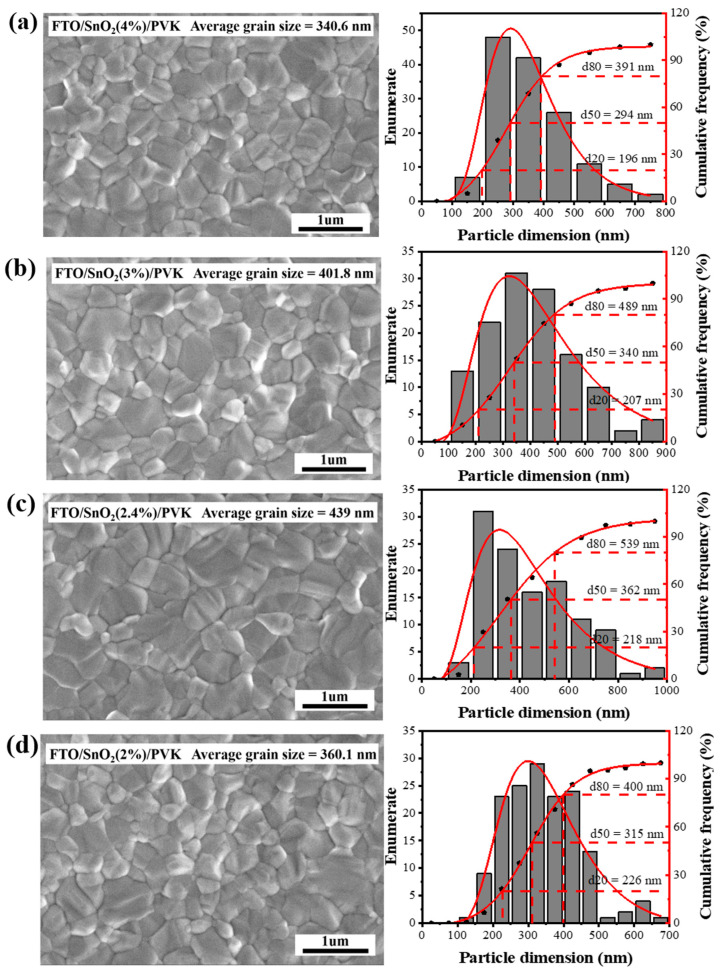
SEM images of perovskite and particle dimension distribution. (**a**) SnO_2_ 4%, (**b**) SnO_2_ 3%, (**c**) SnO_2_ 2.4%, and (**d**) SnO_2_ 2%.

The SnO_2_ hydrocolloidal dispersion solution purchased from the aforementioned company generally exists as a SnO_2_ quantum dot aqueous solution, with an original concentration of 12%. The original SnO_2_ solution may emerge in a cluster state; thus, further dilution of the original solution is necessary. Bonding issues are very important factors determining the quality of deposited perovskite films. As shown in Figure 2, through scanning electron microscopy (SEM), the morphological evolution of the perovskite films deposited on the different SnO_2_ concentrations is evident. When the SnO_2_ concentration was 4%, the grain size of the perovskite film deposited on it was small, with the average grain size being about 340 nm. As the SnO_2_ concentration was decreased to 3%, the grain size of perovskite films increased to 401 nm; the grain size further increased to 439 nm as the concentration was decreased to 2.4%. At this point, the perovskite layer showed a more compact grain boundary and a smooth surface topography, indicating that SnO_2_ at this concentration is more suitable as an ETL for PSC device fabrication. The further dilution (2% SnO_2_) resulted in a rough surface and inferior perovskite deposition. The surface morphologies of different SnO_2_ ETLs were compared, and the differences were not obvious (Supporting Information Figure S1).

**Figure 3:**
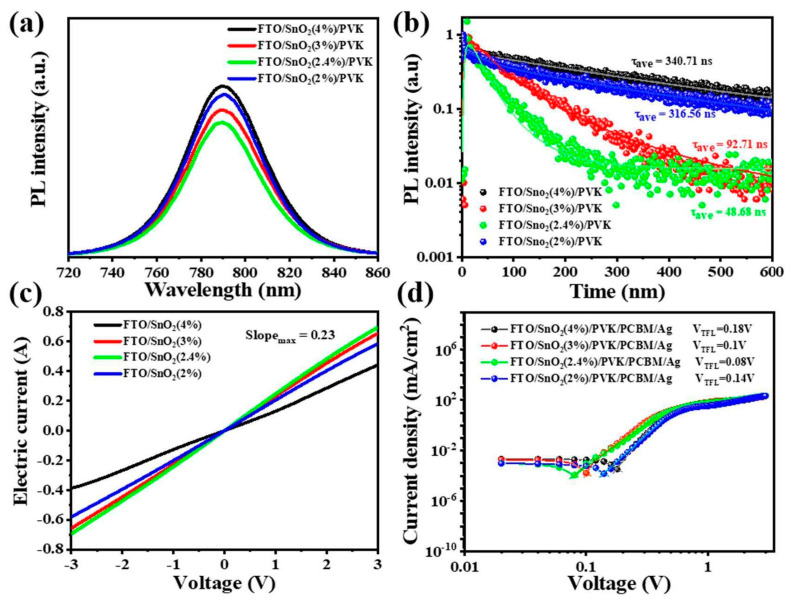
(**a**) PL comparison, (**b**) TRPL, (**c**) conductivity of perovskite films deposited on different SnO_2_ layers and (**d**) SCLC measurement.

We further investigated the steady-state photoluminescence (PL) spectra and time-resolved photoluminescence (TRPL) of perovskite films grown on different concentrations of SnO_2_. The quenched luminous intensity suggested that the electron transport layer had an enhanced capacity to extract and collect charge carriers generated by the perovskite layer. When the concentration of SnO_2_ was 4%, the highest PL intensity indicated carrier accumulation and aggregation in the perovskite film. This also implied that the perovskite layer was not conducive to good carrier transport performance, as confirmed by the conductivity analysis in Figure 3b. As SnO_2_ concentration decreased (3%), the carrier transportation that occurred in the electron transport layer was improved, and the conductivity of the perovskite layer was correspondingly enhanced. As the SnO_2_ was diluted to 2%, the carriers produced by the perovskite film became more favorably absorbed by the electron transport layer, resulting in the lowest corresponding luminous spectral intensity. The quenching efficiencies of PL emission at the interface between the perovskite and ETL were in the order of 2.4% > 3% > 2% > 4%, which indicated more effective electron extraction from the perovskite/diluted SnO_2_ (2.4%).

According to the formula for conductivity (conductivity = current/voltage = 1/resistance), it is evident that a steeper slope corresponds to higher conductivity and lower resistance in the electron transport layer, indicating improved carrier transport efficiency. When the SnO_2_ concentration is 4%, it can be clearly seen that the slope is the lowest, and the value is about 0.13, which indicates that the large resistance generated by SnO_2_ hinders carrier transport. With a decreased SnO_2_ concentration, the perovskite conductivity is obviously increased. When the SnO_2_ concentration is 2.4%, the resulting perovskite film exhibits the highest electrical conductivity (approximately 0.23), confirming minimal resistance and enhanced charge carrier absorption from the perovskite layer. The electron-transport properties of diluted SnO_2_ layers were evaluated using the space charge-limited current (SCLC) measurement taken with the electron-only device, as indicated in Figure 3d. The evaluated trap-filled limit voltages (V_TFL_s) of diluted SnO_2_ with the original, 4%, 3% and 2.4% concentrations are 0.18, 0.1, 0.08 and 0.14 V, respectively. The lower V_TFL_ for SnO_2_ of 2.4% indicates a lower trap density.

**Figure 4:**
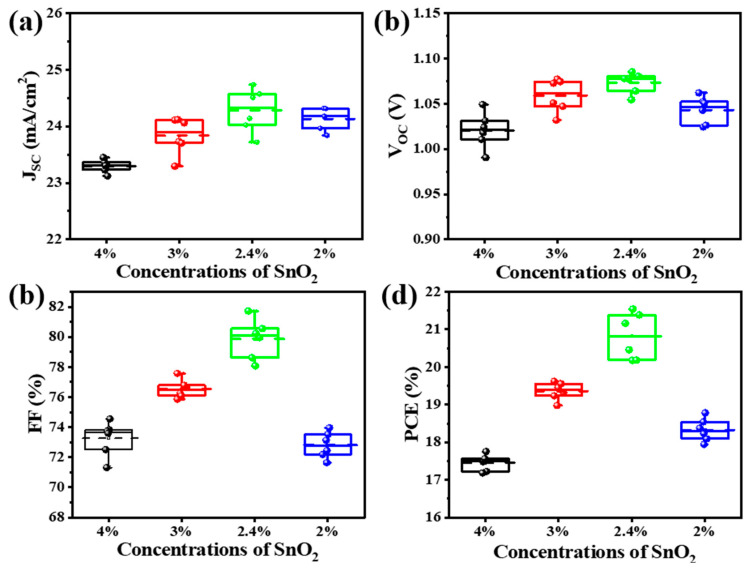
Statistical distribution of the photovoltaic parameters for different PSCs with varied SnO_2_ concentrations. Distribution of (**a**) *J*_sc_, (**b**) Voc, (**c**) FF, and (**d**) PCE.

To more accurately quantify the impact of the SnO_2_ layer, the detailed distribution of the performance parameters extracted from more cells is shown in Figure 4. In detail, when the SnO_2_ concentration was 4%, the device exhibited an average PCE of only 17.40%, a Voc of 1.02 V, an FF of 73.26%, and a *J*_SC_ of 23.29 mA/cm^2^. This can be attributed to the thick SnO_2_ thin film and its inferior perovskite conductivity. When the concentration increased to 3% and 2.4%, the device performance became gradually enhanced. In particular, for the device with an SnO_2_ concentration of 2.4%, it showed the highest average PCE of 20.27%. The further addition of water in SnO_2_ solution (2%) resulted in the inferior performance of device, with an average PCE of only 18.28%. To investigate the effect of different concentrations of SnO_2_ on the performance of perovskite solar cells, the activities of different perovskite solar cells are summarized in Table 1.

The corresponding external efficiency (EQE) spectra and integrated current density (integrated *J*_sc_) values of different devices were recorded; the results indicated that the integrated *J*_sc_ values were in accordance with the *J*_sc_ values from the J–V curves (Supporting Information Figure S2). The enhanced photovoltaic performance can be ascribed to the following factors: (1) dense and uniform coverage of the SnO_2_ layer on the substrate; (2) the smooth interface and controlled defects originating from uniformly dispersed SnO_2_ colloids in the aqueous solution; and (3) improved perovskite crystallinity and conductivity. 

## 4. Conclusions 

In summary, aqueous SnO_2_ solutions with different concentrations were employed in perovskite solar cells. Decreased concentrations facilitated perovskite crystallinity and conductivity, and improved the performance of the devices. The PSC with 2.4% SnO_2_ showed an efficiency of 20.27%. However, the further dilution of SnO_2_ with a 2% concentration resulted in reduced performance. A moderate SnO_2_ concentration is conducive to dense and uniform coverage, conductivity, and the electron transport of the perovskite layer. An appropriate SnO_2_ concentration is essential for efficient perovskite solar cells.

## Figures and Tables

**Table 1:** The performance of perovskite solar cells with different SnO_2_ concentrations.

Concentration Ratios	PCE (%)	V_OC_ (V)	FF (%)	*J*_SC_ (mA/cm^2^)
SnO_2_ (4%)	17.40	1.02	73.26	23.29
SnO_2_ (3%)	19.34	1.06	76.54	23.84
**SnO_2_ (2.4%)**	**20.27**	**1.07**	**79.9**	**24.29**
SnO_2_ (2%)	18.28	1.04	72.82	24.14

## Data Availability

The data that support the findings of this study are available within the article.

## Supplementary Materials

The following supporting information can be download at: https://www.mdpi.com/article/10.3390/nano14171406/s1, Figure S1: Surface morphologies of different SnO_2_ with varied concentration; Figure S2: The EQE spectra and integrated current of different devices.
