# Supplementary material for: SnO2-Based Interfacial Engineering towards Improved Perovskite Solar Cells
Source: Nanomaterials (Basel). 2024 Aug 28;14(17):1406. doi: 10.3390/nano14171406 (PMC11396952; doi:10.3390/nano14171406)
Supplement: Supplementary file 1 [file nanomaterials-14-01406-s001.zip › nanomaterials-3178190-supplementary.pdf]

Supporting Information

SnO_2 based interfacial engineering towards improved perovskite solar cells

Bing'e Li [1], Chuangping Liu [1] and Xiaoli Zhang [1, *]

1 School of Physics and Opto-electronic Engineering, Guangdong Provincial Key Laboratory of Information Photonics Technology, Guangdong University of Technology, Guangzhou 510006, China

* Correspondence: xlzhang@tju.edu.cn; Tel.: +86-020-3932-226

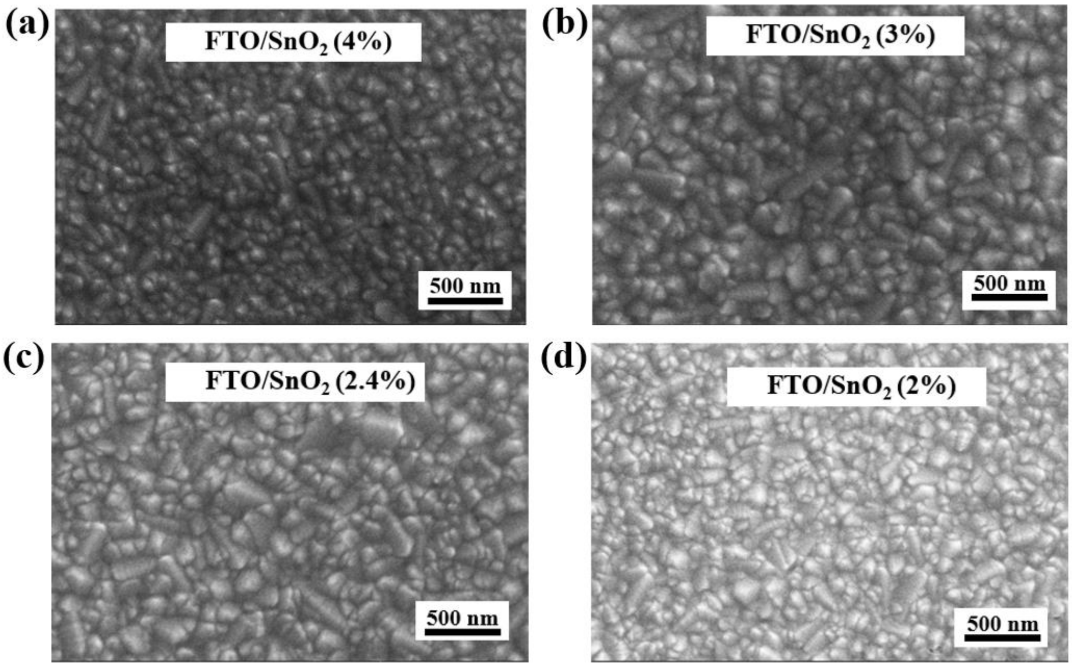

Figure S1. Surface morphologies of different SnO_2 with varied concentration.

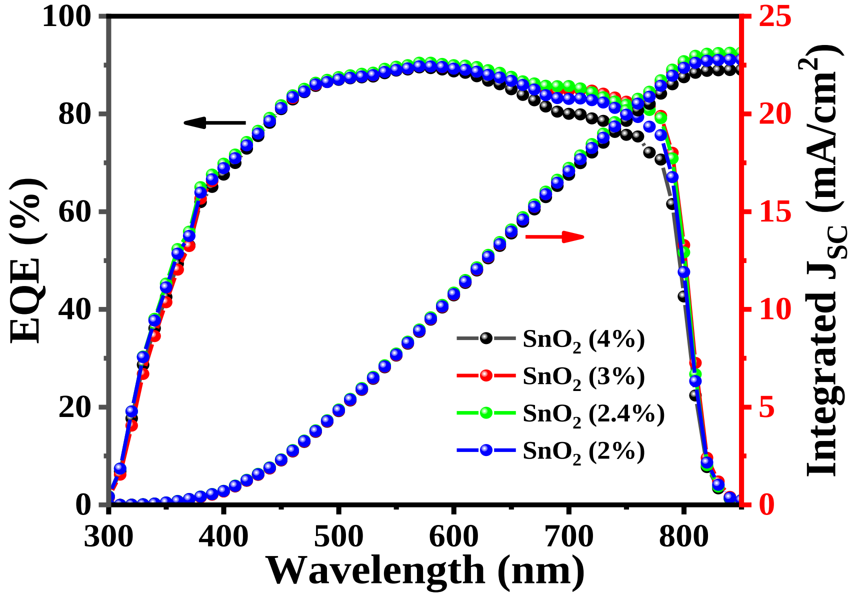

Figure S2. The EQE spectra and integrated current of different devices.
